# Development and validation of a machine learning model for predicting unplanned removal of totally implantable venous access ports in patients with breast cancer

**DOI:** 10.3389/fonc.2026.1780456

**Published:** 2026-03-25

**Authors:** Yinhuan Wang, Xin Wu, Shuang Song, Xiaona Yan, Yi Zhang, Ying Yang, Jing Liu

**Affiliations:** Department of Breast and Thyroid Surgery, First Affiliated Hospital of Army Medical University, Chongqing, China

**Keywords:** breast cancer, machine learning, risk prediction model, totally implantable venous access port, unplanned removal

## Abstract

**Objective:**

Analyze risk factors for unplanned removal of totally implantable venous access ports (TIVAP) in breast cancer patients and develop a machine learning prediction model.

**Methods:**

A single-center, retrospective study was conducted, including breast cancer patients(n=1,258) who underwent TIVAP removal at the Department of Breast and Thyroid Surgery of a tertiary hospital in Chongqing between October 2013 and March 2023.Variable screening was performed using univariate logistic regression and the Least Absolute Shrinkage and Selection Operator (LASSO) method to identify potential risk factors. The variables selected through this process were then incorporated into a multivariate logistic regression for further analysis. The dataset was randomly split into a training set (n=881) and a validation set (n=377) in a 7:3 ratio. Subsequently, four machine learning prediction models—random forest, decision tree, logistic regression, and XGBoost—were developed and evaluated. The model demonstrating the best performance was selected as the final predictive model.

**Results:**

The XGBoost model developed in this study demonstrated optimal performance in predicting the risk of unplanned removal of totally implantable venous access ports (TIVAP) in breast cancer patients. In the training set, the area under the receiver operating characteristic curve (AUC) was 0.826 (95% CI: 0.783–0.869), with a specificity of 0.829 and a sensitivity of 0.683. In the validation set, the AUC was 0.751 (95% CI: 0.704–0.839), with a specificity of 0.820 and a sensitivity of 0.636, indicating that the model possesses strong discriminative ability and robust predictive performance. Furthermore, the study identified independent risk factors for unplanned TIVAP removal, primarily including body mass index (BMI), TNM stage, implantation route, number of successful puncture attempts, coagulation function indices, neutropenia, and catheter indwelling time. The calibration curve shows that the XGBoost model is well calibrated and fitted, and DCA indicates that the model has good clinical net benefits.

**Conclusion:**

This study developed a machine learning prediction model for the risk of unplanned removal of totally implantable venous access ports (TIVAP) in breast cancer patients. The model demonstrated favorable discrimination and calibration, with robust predictive performance. It contributes to the early identification of high-risk patients in clinical practice, enabling targeted interventions and preventive measures to reduce the incidence of unplanned TIVAP removal.

## Introduction

According to 2024 statistics from the American Cancer Society (ACS), breast, lung, and colorectal cancers collectively account for 51% of all new cancer cases in female patients. With breast cancer constitutes 32% of female cancers, establishing it as the most common malignancy among women (1). Adjuvant therapy is an important part of comprehensive systemic therapy for breast cancer patients, with intravenous chemotherapy serving as its primary method (2). According to the different staging of breast cancer, patients require 6 to 8 cycles of neoadjuvant or adjuvant therapy, typically completed over 4-6 months. For patients who experience chemotherapy-related adverse effects leading to treatment prolongation, suboptimal antitumor response, or advanced-patients who require long-term maintenance therapy, the treatment duration may extend from 1 to 3 years. Therefore, the choice of intravenous access is particularly important for patients undergoing systemic therapy for breast cancer (3). Given the high concentration and strong irritancy of chemotherapeutic drugs, coupled with the prolonged chemotherapy cycles in breast cancer, repeated venipunctures may lead to vascular injury (4, 5).

The totally implantable venous access port (TIVAP) is a fully implantable closed venous access system that can be left in the body for a long period of time. It can avoid repeated venipunctures, help to protect peripheral vessels, prevent drug extravasation and reduce patient discomfort. Meanwhile, its advantages of portability, simple maintenance and unrestricted by daily activities which has become the preferred intravenous access for many tumor patients globally (6–8). unplanned removal refers to the early removal of TIVAPs due to human factors, or complications. This shortens the duration of the TIVAPs, affects the treatment outcomes and quality of life, while increasing patient suffering and financial burden (9, 10).

Currently, there has been analysis of risk factors for unplanned removal of TIVAP in cancer chemotherapy patients, but research on constructing machine learning-based risk prediction models is limited, with small sample sizes and a primary focus on unplanned removal in patients with hematologic malignancies. Therefore, this study aims to analyze the risk factors for unplanned removal of TIVAP in breast cancer patients and to develop and validate a machine learning-based prediction model. The goal is to provide a basis for clinical healthcare professionals to early identify patients at risk of TIVAP unplanned removal and to implement preventive and therapeutic measures in a timely manner.

## Methods

### Study population

**Figure 1:**
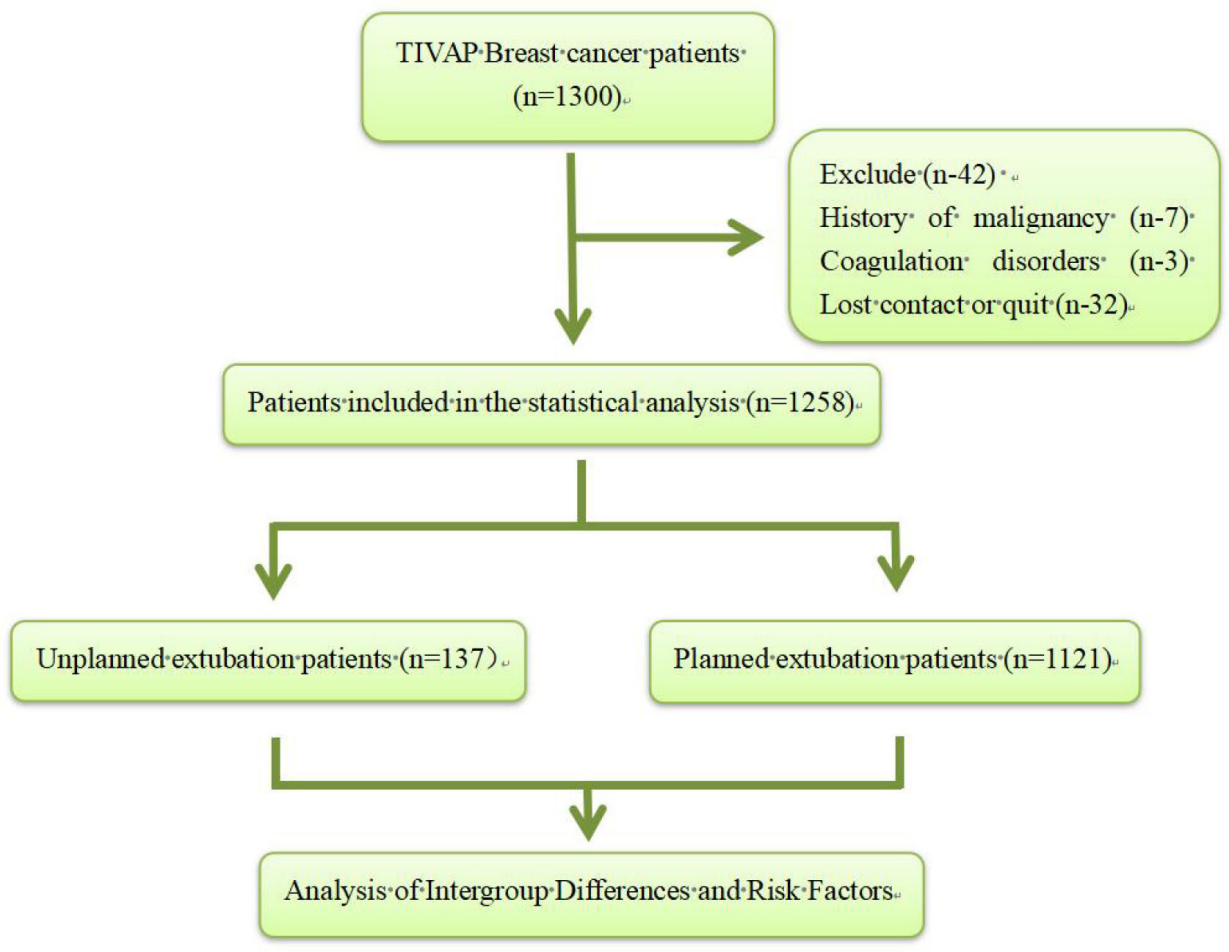
Research flowchart.

This study is a retrospective cohort study that included 1,300 breast cancer patients who underwent TIVAP extubation from October 2013 to March 2023. Patients are female, aged between 18 and 70, with breast cancer who require chemotherapy and need to undergo TIVAP. And all patients have normal cognition with basic communication and comprehension abilities. Exclusion criteria include a history of other malignant tumors, severe cardiopulmonary or brain disease, coagulation disorders, confirmed or suspected infections (including bacteremia and sepsis), as well as poor patient compliance and withdrawal from the study cohort. The research workflow is depicted in **Figure 1**, and the study received ethical approval from the Ethics Committee of the First Affiliated Hospital of Army Medical University.(KY2023136). This study was performed in line with the principles of the Declaration of Helsinki.

### Data collection

Research data were collected from the electronic medical record system. Including age, body mass index (BMI), underlying diseases, TNM stage, pathological classification, chemotherapy regimen, implantation route, TIVAP implantation side, catheter tip position, number of puncture attempts, first use time, coagulation function, catheter indwelling time, and neutrophil counts, etc. Patients who experience complications during TIVAP placement and require unplanned removal are managed by the TIVAP management team in conjunction with a multidisciplinary consultation, and a specialist is assigned to follow up and collect data. All data is entered and then double-checked by two people. If there are discrepancies in the data, they are identified and corrected to ensure accuracy (**Table 1**).

**Table 1:** Determination of unplanned removal due to complications.

Cause	Determination criteria
Local Infection	a. Localized redness, swelling, pain, induration, and pus accumulation at the port insertion site and puncture site. b. Laboratory tests reveal exudate and pus, and positive wound swab culture results, indicating local infection.
Systemic Infection	a. Both the port chamber and peripheral venous blood cultures are positive and the microorganisms are identical; b. Clinical manifestations (fever >38 °C, chills, hypotension) are present with no alternative source of bacteremia.
Catheter-related Thrombosis	a. Edema of the arm, neck, and face on the side of the port placement, increased skin temperature, and pain in the affected limb. b. Vascular ultrasound demonstrates thrombus formation.
Catheter Occlusion	a. Infusion flow rate is absent or significantly reduced. b. No blood or slow blood return when withdrawing, fluid leakage at the puncture site or inability to flush the tube. c. Other complications have been excluded.
Port inversion	a. The bottom of the port seat is in direct contact with the skin. Palpation reveals an enlarged area that is hard, with no sense of layers and unclear boundaries. b. Non-coring needle fails to access the septum.

### Statistical analysis

Data processing and statistical analyses were performed using SPSS 26.0 (IBM Analytics, USA) and R (version 4.4.3; R Foundation for Statistical Computing, Vienna, Austria). Normally distributed continuous variables were expressed as mean ± standard deviation, while categorical variables were reported as frequencies (%). Intergroup comparisons utilized χ² tests or Pearson’s χ² tests, with Fisher’s exact test applied when appropriate. Univariate analysis was performed for each variable, with a p-value < 0.05 considered statistically significant. Variable selection to identify risk factors was conducted using the Least Absolute Shrinkage and Selection Operator (LASSO) method. The dataset was then randomly split into a training set and a validation set at a 7:3 ratio. Based on the identified predictors, four machine learning prediction models were constructed using Random Forest, Decision Tree, Logistic Regression, and XGBoost. Model performance was evaluated using receiver operator characteristic (ROC) curves and calibration curves, with the optimal model was selected. Given the relative imbalance in the distribution of the outcome variable, we applied a class weighting method during the machine learning process to address this potential bias Use the area under curve (AUC) to assess discrimination. Use calibration curves to assess calibration ability, The clinical utility of the optimal model was determined by evaluating its net benefit across a range of threshold probabilities using decision curve analysis (DCA).

## Results

Clinical data were collected from 1,258 patients with breast cancer who underwent TIVAP removal. Among these, 1,121 cases (89.11%) underwent planned extubation without complications after treatment completion or when no longer clinically indicated, while the 137 cases (10.89%) involved complications and unplanned removal. According to the criteria in **Table 1**, we analyzed the data of 137 patients who experienced complications and underwent unplanned removal. The main causes were local infection (24 cases, 1.91%), catheter-related factors (15 cases, 1.19%), and thrombosis (79 cases, 6.28%).

### Univariate analysis

A total of 1,258 breast cancer patients who underwent TIVAP extubation were divided into planned extubation group and unplanned removal group. Compared with unplanned removal group, the planned extubation group had significant differences in age, BMI, TNM stage, implantation route, number of puncture attempts, coagulation function, catheter indwelling time, and neutropenia (p < 0.05). This suggests that the above factors may be associated with complications and unplanned removal in breast cancer patients with TIVAP implantation. There was no significant difference observed between the two groups in terms of underlying diseases. Pathological classification, chemotherapy regimens, TIVAP implantation side, catheter tip position, and first use time (p > 0.05) (**Table 2****).**

**Table 2:** Univariate analysis of unplanned removal due to complications in TIVAP implantation.

Factor	Unplanned removal group (n=137)	Planned extubation group (n=1121)	χ^2^	*P*
**Age (years)**			**3.875**	**0.049**
> 55	37 (27.01%)	222 (19.80%)		
≤ 55	100 (72.99%)	899 (80.20%)		
**BMI (**kg/m^2^)			**7.730**	**0.005**
> 24	73 (53.28%)	458 (40.86%)		
≤ 24	64 (46.72%)	663 (59.14%)		
**Underlying diseases**			**1.464**	**0.226**
No	115 (83.94%)	982 (87.60%)		
YES	22 (16.06%)	139 (12.40%)		
**TNM stage**			**12.557**	**<0.001**
I-II	91 (66.42%)	893 (79.66%)		
III-IV	46 (33.58%)	228 (20.34%)		
**Pathological classification**			**5.036**	**0.169**
Her-2 positive	25 (18.25%)	182 (16.24%)		
Luminal A	30 (21.90%)	323 (28.81%)		
Luminal B	47 (34.31%)	402 (35.86%)		
Tripple-negative	35 (25.55%)	214 (19.09%)		
**Chemotherapy regimens**			**2.820**	**0.244**
EC-T	71 (51.82%)	627 (55.93%)		
TE/TEL	57 (41.61%)	452 (40.32%)		
Others	9 (6.57%)	42 (3.75%)		
**Implantation route**			**28.547**	**<0.001**
Internal Jugular Vein	57 (41.61%)	237 (21.14%)		
Upper Arm Vein	80 (58.39%)	884 (78.86%)		
**TIVAP implantation side**			**0**	**0.985**
Right	71 (51.82%)	580 (51.74%)		
Left	66 (48.18%)	541 (48.26%)		
**Catheter tip position**			**0.488**	**0.485**
T5~T7	127 (92.70%)	1019 (90.90%)		
Others	10 (7.30%)	102 (9.10%)		
**Number of puncture attempts**			**5.979**	**0.014**
Once	97 (70.80%)	895 (79.84%)		
Twice or more	40 (29.20%)	226 (20.16%)		
**First use time (hours)**			**2.469**	**0.116**
<12	104 (75.91%)	778 (69.40%)		
≥12	33 (24.09%)	343 (30.60%)		
**Coagulation function**			**50.723**	**<0.001**
Normal	93 (74.45%)	1003 (89.30%)		
Abnormal	44 (25.55%)	118 (10.70%)		
**Catheter indwelling time (months)**			**49.113**	**<0.001**
<6	70 (51.09%)	260 (23.19%)		
≥6	67 (48.91%)	861 (76.81%)		
**Neutropenia**			**9.197**	**0.002**
Yes	21 (15.33%)	86 (7.67%)		
No	116 (84.67%)	1035 (92.33%)		

Univariate analysis of all patients with unplanned removal of TIVAP.

### Screening for risk factors for unplanned removal of TIVAP

**Figure 2:**
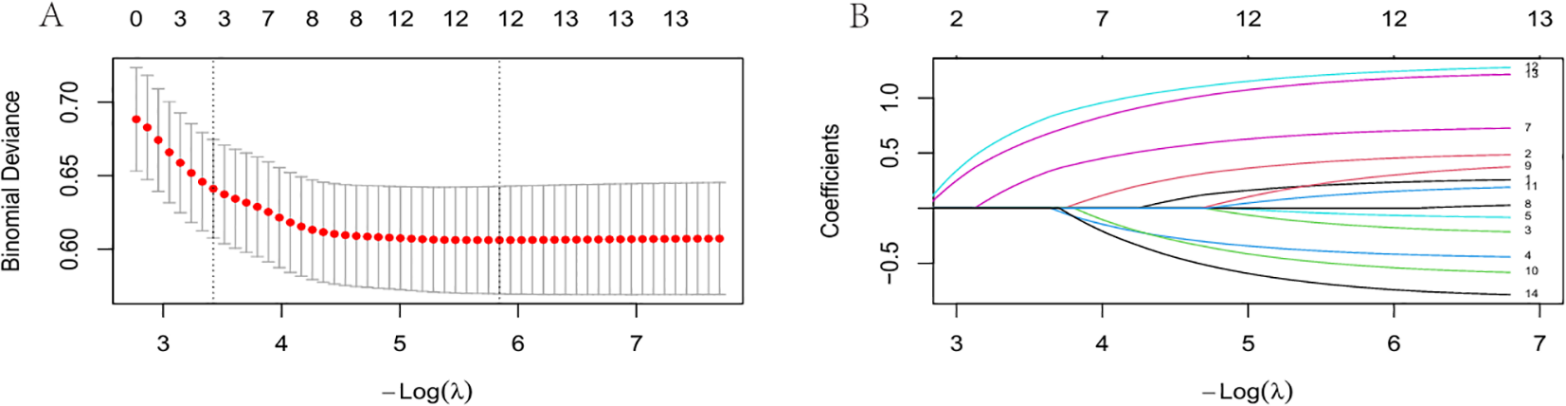
**(A)** Cross-validation results. The region between the two dashed vertical lines indicates values of log(λ) within standard deviation. The left dashed line corresponds to the λ value yielding the minimal error. At log(λ) = 0.003, 13 variables were selected. **(B)** Distribution of LASSO coefficients for 13 variables. A vertical line is drawn at the λ value selected by 10-fold cross-validation. As λ decreases, the regularization strength increases, enhancing the model’s variable selection capability.

Variable screening via LASSO regression selected 13 predictors with non-zero coefficients, including age, BMI, underlying diseases, TNM stage, pathological classification, implantation route, implantation side, catheter tip position, number of puncture attempts, first use time, coagulation function, catheter indwelling time, and neutropenia (**Figure 2**).

### Logistic regression analysis for unplanned removal of TIVAP

Were performed using univariate analysis and LASSO regression to identify risk factors. Subsequently, multivariate logistic regression analysis was conducted on the selected variables. This analysis confirmed that BMI, TNM stage, implantation route, number of successful puncture attempts, coagulation function, catheter indwelling time, and neutropenia were independent risk factors for unplanned TIVAP extubation (all P < 0.05) (**Table 3**).

**Table 3:** Logistic regression analysis for unplanned removal of TIVAP.

Risk factor	β	SE	Wald χ^2^	P	OR(95%CI)
Constant	-2.785	0.235	140.084	<0.001	0.062
BMI	0.452	0.190	5.660	0.017	1.572(1.083-2.281)
TNM stage	0.519	0.207	6.292	0.012	1.681(1.120-2.522)
Implantation route	0.814	0.199	16.654	<0.001	2.257(1.527-3.336)
Number of puncture attempts	0.565	0.213	7.037	0.008	1.760(1.159-2.672)
Coagulation function	1.166	0.231	25.571	<0.001	3.208(2.042-5.041)
Catheter indwelling time	0.979	0.213	21.129	<0.001	2.662(1.754-4.042)
Neutropenia	0.759	0.278	7.411	0.006	2.136(1.237-3.688)

The reference group for the implantation route was the upper arm vein.

### Machine learning model selection

**Figure 3:**
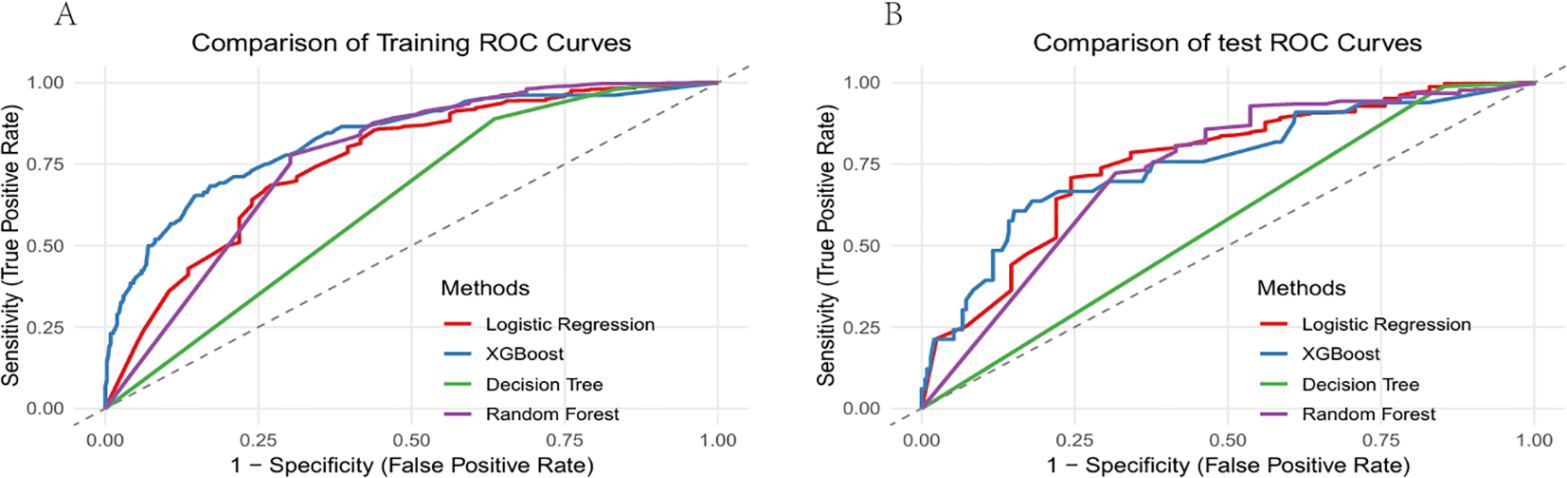
**(A)** ROC curves for training sets of different machine learning models. **(B)** ROC curves for validation sets of different machine learning models.

The 7 independent risk factors identified via multivariate logistic regression-BMI, TNM stage, implantation route, number of puncture attempts, coagulation function, catheter indwelling time, and neutropenia-served as input features for the machine learning models. Four distinct machine learning prediction models—Random Forest, Decision Tree, Logistic Regression, and XGBoost. To optimize predictive performance, a systematic grid search was conducted to tune the key hyperparameters for each algorithm. These models were then benchmarked to compare their predictive performance. As shown in **Table 4**, the benchmark results present various performance metrics for each model, including the area under the ROC curve (AUC), sensitivity, and specificity. **Figure 3** displays the ROC curves of the four models, providing a graphical representation of their predictive ability. To determine the best-performing model, AUC values were used as the primary criterion, and the models were ranked accordingly. The XGBoost model achieved the highest predictive performance, with results on the training set (AUC = 0.826, specificity = 0.829, sensitivity = 0.683) and on the validation set (AUC = 0.751, specificity = 0.820, sensitivity = 0.636). Consequently, the XGBoost model was selected as the final predictive model.

**Table 4:** AUC values for each machine learning model (95%CI).

Dataset allocation	Decision tree	Random forest	Logistic regression	XGBoost
Training sets	0.633 (0.582-0.683)	0.767 (0.715-0.821)	0.761 (0.701-0.814)	0.826 (0.783-0.869)
Validation sets	0.569 (0.502-0.642)	0.733 (0.643-0.814)	0.757 (0.706-0.813)	0.751 (0.704-0.839)

**Figure 4:**
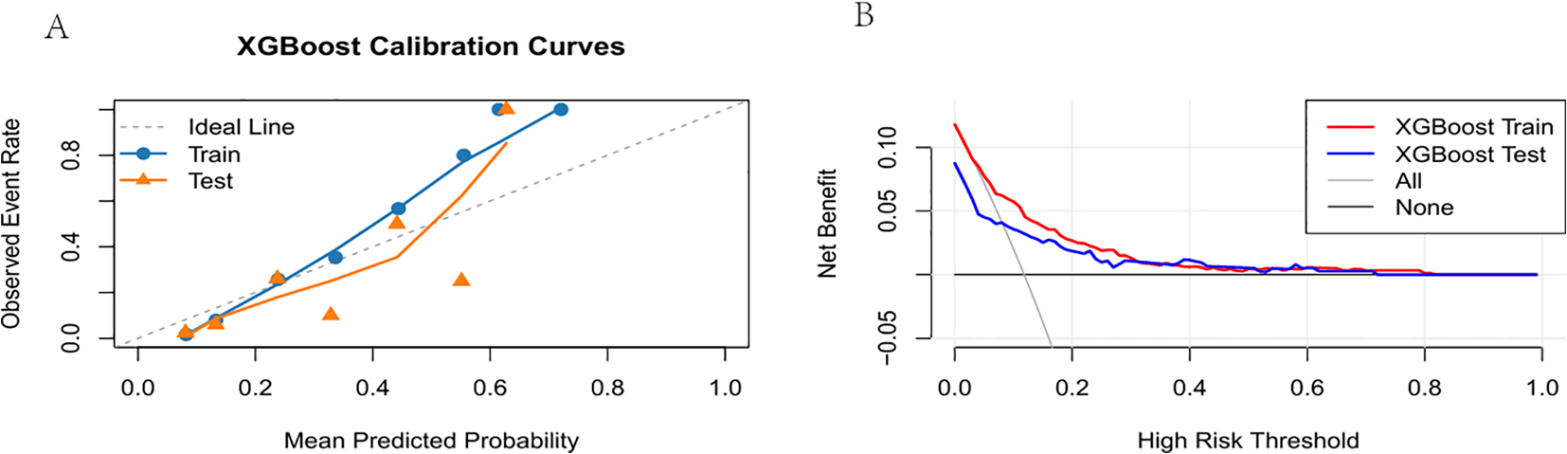
**(A)** Calibration curve, **(B)** Decision curve.

The predictive performance of the XGBoost model was assessed using calibration curves and decision curve analysis (DCA). As shown in (**Figure 4A**), the calibration curve demonstrated showed good agreement between the predicted probabilities and the observed outcomes. DCA was performed to assess the clinical utility of the XGBoost model (**Figure 4B**). The model maintained non-negative net benefit over the full threshold range of 0–1.0 in training and test sets, with highly consistent curves and no overfitting, indicating stable predictive performance. Significantly positive net benefit was observed within the threshold range of 0.0–0.6, where model-based risk stratification and targeted interventions outperformed both no-intervention and universal intervention strategies. Universal anti-extubation measures were only marginally beneficial at thresholds < 0.1, after which net benefit fell rapidly into negative territory, suggesting wasteful resource use and unnecessary harm to low-risk patients. Given the comparable AUC values of Logistic regression and XGBoost in the validation set, we further generated the calibration curve and decision curve for Logistic regression (**Supplementary Figure 5**). The results demonstrated that Logistic regression exhibited inferior calibration performance and a narrower range of clinical utility in the decision curve analysis compared to XGBoost. Therefore, XGBoost was selected as the optimal model. These results validate that the XGBoost model permits accurate risk stratification for unplanned removal with strong clinical applicability.

## Discussion

Chemotherapy plays a crucial role in the comprehensive treatment of breast cancer, typically involving 6 to 8 cycles over a period of 4 to 6 months. In some cases, patients may undergo targeted therapy in combination with chemotherapy, extending the treatment duration to 1 year or longer (11). TIVAP is widely used in clinical practice due to its safety and high utilization rate (12), However, during TIVAP implantation or placement, there are many factors that can lead to unplanned removal, increasing patient suffering and economic costs, and affecting the physical and mental health of patients and the effectiveness of treatment. Therefore, this study developed predictive models for TIVAP extubation risk using multiple machine learning algorithms, with comparative performance analysis identifying the optimal model. The optimal machine learning model for predicting unplanned TIVAP extubation has good discriminative ability and potential clinical practicality, and is significantly correlated with risk stratification of unplanned removal. In addition, the model exhibits good generalization ability. This approach aims to provide clinical guidance on the risk factors influencing TIVAP unplanned removal, thereby reducing complication rates, ensuring TIVAP safety, and enhancing patients’ confidence in treatment.

Common complications of TIVAP include infection, thrombosis, catheter malposition/dislodgement/fracture, and port exposure (13). The overall incidence of complications ranges from 9.6% to 38.0% (14, 15). In our study, 137 out of 1,258 patients experienced complications leading to unplanned removal, resulting in an incidence rate of 10.89%, which falls within the range reported in previous studies. Although some studies have shown that TIVAP has a lower infection rate than other infusion devices and can serve as a permanent venous access for cancer patients (12), it also carries postoperative complication. Infection is the most common complication, not only prolonging the patient’s recovery time but also affecting their quality of life (16).

In our TIVAP extubation risk prediction model identified seven critical predictors: neutropenia, BMI, coagulation function, TNM stage, catheter indwelling time, implantation route, and number of puncture attempts. Neutropenia is considered a risk factor for infection because it causes intestinal bacterial translocation, increasing microbial load in the bloodstream. Evan et al. (17) confirmed neutropenia as a primary risk factor for catheter-related infections, while Zerati’s study of 1,255 TIVAP implantations reported significantly higher infection rates in neutropenic patients (n=92) versus non-neutropenic controls (18). These findings are consistent with our study that neutropenia is one of the risk factors for complications leading to unplanned removal in patients.

Catheter-related thrombosis is a common complication of catheter placement, with an incidence rate of 2% to 30% (18). Studies report obese patients face a 2-3-fold higher risk of venous thromboembolism than normal-weight individuals, and that the risk increases with BMI (19, 20). Our findings identify BMI>24 kg/m² as a significant risk factor for unplanned TIVAP extubation. Liu (21) et al. found that obesity is an independent risk factor for unplanned TIVAP extubation in breast cancer patients, consistent with the findings of our study. Obesity is a prothrombotic condition characterized by increased expression of prothrombotic molecules such as tissue factor and plasminogen activator inhibitor-1 (PAI-1), systemic inflammation, and enhanced platelet activation (22). It induces vascular endothelial dysfunction and promotes a hypercoagulable milieu (23). In cancer patients, pre-existing hemodynamic hypercoagulability—combined with vascular injury during catheter insertion—readily activates coagulation cascades, leading to localized thrombotic occlusion. Clinicians must prioritize patient education on weight management to mitigate thrombosis risk.

Additionally, elevated venous pressure from patient activity, increased intrathoracic pressure, or improper flushing techniques may cause blood reflux into the catheter, forming occlusive clots (24).Higher TNM stages correlate with greater thrombosis risk, potentially linked to elevated Factor V levels and prothrombin gene mutations in advanced cancer patients (25). Prolonged stimulation of the vascular wall by the catheter and chemotherapy drugs can damage vascular endothelial cells, leading to aggregation of platelets, red blood cells, and damaged endothelial cells, thereby causing thrombosis (26). Therefore, the longer the chemotherapy cycle and the duration of TIVAP placement, the greater its risk of thrombosis, This is consistent with the results of this study.

Tippit’s (27) retrospective analysis of 297 breast cancer patients with TIVAPs revealed a 5-fold higher symptomatic thrombosis rate in arm-ports versus chest-ports (P<0.05). This elevated risk in arm port is mechanistically linked to catheter-to-vein diameter ratios exceeding 45%, where thrombosis risk escalates when catheters occupy >50% of the vascular lumen (28)—validating our finding that upper arm vein port route constitutes an independent risk factor for unplanned removal. Repeated puncture attempts cause progressive venous endothelial damage, triggering platelet aggregation, coagulation cascades, and inflammatory responses that alter hemodynamics and hemorheology, ultimately increasing thrombosis risk, consistent with the findings of Li et al (29, 30). While some studies employ risk scoring systems to assess logistic model (31), our study included the catheter tip position—which showed no statistically significant difference in univariate analysis—in the multivariate logistic regression analysis model to evaluate its validity. The results were consistent with those of the univariate analysis, indicating the validity of the multivariate regression analysis model. We found that prolonged TIVAP indwelling time is one of the risk factors for unplanned removal, corroborating Jia et al.’s cohort study (32).

Furthermore, our study found that the primary causes of unplanned removal of TIVAPs in breast cancer patients were infection, catheter occlusions, or thrombosis. Healthcare providers strictly adhere to aseptic techniques during TIVAP implantation and postoperative maintenance, and provide health education to prevent and reduce the incidence of infection. Before TIVAP implantation, select an appropriate vessel for implantation under ultrasound guidance and avoid repeated punctures and catheter insertion. Ensure that the ratio of catheter diameter to vessel diameter is ≤45% to reduce the incidence of thrombosis. Meanwhile, encourage patients to use non-pharmacological methods to prevent thrombosis postoperatively, including early limb mobilization and exercise after TIVAP implantation, maintaining daily activities, adequate hydration, and weight management. If thrombosis is detected via ultrasound, anticoagulant therapy should be initiated promptly. Regular maintenance of the TIVAP catheter during the placement period (4 weeks) to reduce the incidence of catheter occlusions. Educate patients and their families on relevant medical knowledge so that they can learn the correct methods of port care which will reduce complications.

### Limitations

Limitations of this study: First, the single-center retrospective design and limited sample size may introduce selection bias. Second, the absence of external dataset validation constrains the generalizability of the findings. Future research should aim to validate the model in larger, multi-center, prospective cohorts to enhance its robustness and promote the development of more reliable predictive tools. Additionally, this study was conducted within a single-disease cohort of breast cancer patients. To enhance the generalizability of our findings, future investigations should validate these risk factors in broader populations, including patients with other malignancies or non-oncological conditions requiring TIVAP implantation.

## Conclusion

This study employed machine learning algorithms to develop a predictive model, which significantly enhanced both the predictive efficacy and accuracy of the model. Utilizing this model, we were able to identify high-risk factors associated with the unplanned removal of totally implantable venous access ports and formulate targeted preventive strategies accordingly, thereby reducing related complications and improving patients’ quality of life. During the predictor screening phase, the study integrated univariate logistic regression with the Least Absolute Shrinkage and Selection Operator for preliminary variable screening, and subsequently incorporated the identified risk factors into multivariate logistic regression analysis. This multi-stage screening process resulted in the construction of a more concise and accurate predictive model. Integrating this model into clinical practice can strengthen the management and care of patients with implanted ports, thereby more effectively preventing and reducing the occurrence of unplanned removal events.

## Data Availability

The raw data supporting the conclusions of this article will be made available by the authors, without undue reservation.
