# Supplementary figures and images for: Development and validation of a machine learning model for predicting unplanned removal of totally implantable venous access ports in patients with breast cancer
Source: Front Oncol. 2026 Mar 25;16:1780456. doi: 10.3389/fonc.2026.1780456 (PMC13056808; doi:10.3389/fonc.2026.1780456)

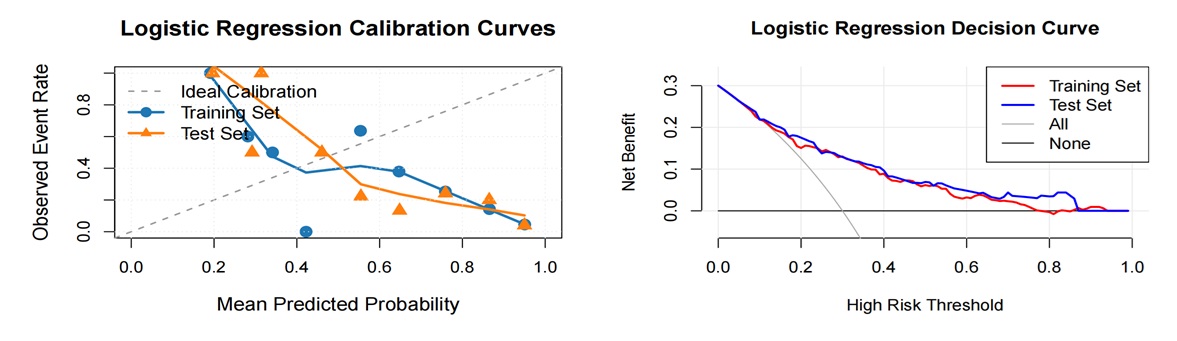

Supplement: Supplementary file 1 [file Image1.jpeg]
